# Diagnosis and management of urinary tract infections in children aged 2 months to 3 years in the Italian emergency units: the ItaUTI study

**DOI:** 10.1007/s00431-022-04457-0

**Published:** 2022-04-06

**Authors:** Francesca Cenzato, Gregorio P. Milani, Angela Amigoni, Francesca Sperotto, Mario G. Bianchetti, Carlo Agostoni, Giovanni Montini, on behalf of the ItaUTI study group, Giovanni Farello, Francesco Chiarelli, Rita Greco, Franco Di Lollo, Fabio Rocco Forte, Sergio Manieri, Luigi Carpino, Mimma Caloiero, Anastasia Cirisano, Salvatore Braghò, Roberto Della Casa, Felice Nunziata, Carmine Pecoraro, Rosario Pacifico, Marcello Lanari, Chiara Ghizzi, Laura Serra, Marcello Stella, Giuseppe Maggiore, Roberto Fiorini, Icilio Dodi, Andrea Morelli, Lorenzo Lughetti, Andrea Cella, Gianluca Vergine, Alessandro De Fanti, Danica Dragovic, Daniele Santori, Giorgio Cozzi, Paola Cogo, Marilena Raponi, Riccardo Lubrano, Mauro de Martinis, Antonio Gatto, Maria Antonietta Barbieri, Antonino Reale, Giorgio Bracaglia, Emanuela Piccotti, Riccardo Borea, Alberto Gaiero, Laura Martelli, Alberto Arrighini, Paola Cianci, Claudio Cavalli, Leonardina De Santis, Benedetta Chiara Pietra, Andrea Biondi, Marco Sala, Laura M. Pogliani, Simonetta Cherubini, Marta Bellini, Paola Bruni, Giovanni Traina, Paola Tommasi, Paolo Del Barba, Sergio Arrigoni, Filippo M. Salvini, Luca Bernardo, Giuseppe Bertolozzi, Silvia Fasoli, Gian Luigi Marseglia, Emilio Palumbo, Annalisa Bosco, Gianpaolo Mirri, Elisabetta Fabiani, Ermanno Ruffini, Luisa Pieragostini, Martina Fornaro, Gabriele Ripanti, Donnina Pannoni, Felici Enrico, Anna Perona, Eleonora Tappi, Oscar Nis Haitink, Ivana Rabbone, Pina Teresa Capalbo, Antonio Urbino, Andrea Guala, Gianluca Cosi, Maria Gabriella Barracchia, Baldassarre Martire, Fabio Cardinale, Fulvio Moramarco, Carmelo Perrone, Angelo Campanozzi, Valerio Cecinati, Alessandro Canetto, Ciro Clemente, Antonio Cualbu, Fabio Narducci, Giuseppina Mula, Pasquale Bulciolu, Roberto Antonucci, Giuseppe Gramaglia, Giuseppe Cavaleri, Carmelo Salpietro, Giovanni Corsello, Rosario Salvo, Marcello Palmeri, Maria Assunta Vitale, Ambra Morgano, Susanna Falorni, Diego Peroni, Stefano Masi, Alessio Bertini, Angelina Vaccaro, Pierluigi Vasarri, Petra Reinstadler, Massimo Soffiati, Maurizio Stefanelli, Alberto Verrotti di Pianella, Catherine Bertone, Stefano Marzini, Liviana Da Dalt, Simone Rugolotto, Floriana Scozzola, Luca Ecclesio Livio, Mauro Cinquetti, Davide Silvagni, Massimo Bellettato

**Affiliations:** 1 Pediatric Emergency Department, Fondazione IRCCS Ca’ Granda Ospedale Maggiore Policlinico, Milan, Italy; 2 Department of Clinical Sciences and Community Health, Università Degli Studi Di Milano, Milan, Italy; 3 Pediatric Intensive Care Unit, Department of Women’s and Children’s Health, University Hospital of Padua, Padua, Italy; 4 Family Medicine Institute, Faculty of Biomedical Sciences, Università Della Svizzera Italiana, Lugano, Switzerland; 5 Pediatric Nephrology, Dialysis and Transplant Unit, Fondazione IRCCS Ca’ Granda Ospedale Maggiore Policlinico, Milan, Italy; 6 Presidio Ospedaliero “San Salvatore” - Clinica Pediatrica Università dell’Aquila, L’Aquila, Italy; 7 Department of Paediatrics, University of Chieti, Chieti, Italy; 8 Presidio Ospedaliero “S. Spirito” Pescara - UOC Pediatria di Pescara, Pescara, Italy; 9 Ospedale “Giuseppe Mazzini”, Teramo, Italy; 10 Presidio Ospedaliero “Madonna delle Grazie”, Matera, Italy; 11 Ospedale San Carlo, Potenza, Italy; 12 Ospedale Annunziata, Cosenza, Italy; 13 Ospedale Giovanni Paolo II, Lamezia Terme, Italy; 14 Ospedale S. Giovanni di Dio, Crotone, Italy; 15 Presidio Ospedaliero “G. Jazzolino”, Vibo Valentia, Italy; 16 Azienda Ospedaliera San Pio, Benevento, Italy; 17 Ospedale Sant’Anna e San Sebastiano, Caserta, Italy; 18 Azienda Ospedaliera Pediatrica Santobono-Pausilipon, Naples, Italy; 19 Azienda Ospedaliera Universitaria S.Giovanni e Ruggi, Salerno, Italy; 20 IRCCS-Policlinico universitario di Sant’Orsola, Bologna, Italy; 21 Ospedale Maggiore Bologna, Bologna, Italy; 22 Ospedale Santa Maria della Scaletta, Imola, Italy; 23 Ospedale Bufalini-Marconi, Cesena, Italy; 24 Ospedale Sant’Anna, Ferrara, Italy; 25 Ospedale di Fidenza, Fidenza, Italy; 26 Ospedale dei bambini “Pietro Barilla” - Azienda Ospedaliero Universitaria di Parma, Parma, Italy; 27 Ospedale Santa Maria delle Croci, Ravenna, Italy; 28 Policlinico di Modena, Modena, Italy; 29 Ospedale Guglielmo da Saliceto, Piacenza, Italy; 30 Ospedale degli Infermi, Rimini, Italy; 31 Arcispedale Santa Maria Nuova, Reggio Emilia, Italy; 32 Ospedale San Polo, Monfalcone, Italy; 33 Ospedale Santa Maria degli Angeli, Pordenone, Italy; 34 IRCCS Materno Infantile Burlo Garofolo, Trieste, Italy; 35 Ospedale Santa Maria della Misericordia, Udine, Italy; 36 Presidio Ospedaliero di Cassino, Frosinone, Italy; 37 Ospedale Santa Maria Goretti, Latina, Italy; 38 Ospedale San Camillo de Lellis, Rieti, Italy; 39 Policlinico Universitario Agostino Gemelli, Rome, Italy; 40 Ospedale Bambino Gesù Palidoro, Rome, Italy; 41 Ospedale Pediatrico Bambin Gesù, Rome, Italy; 42 Ospedale Belcolle, Viterbo, Italy; 43 IRCCS G. Gaslini, Genua, Italy; 44 Ospedale Civile di Imperia, Imperia, Italy; 45 Ospedale San Paolo / Santa Corona Pietra Ligure, Savona, Italy; 46 Ospedale Papa Giovanni XXIII, Bergamo, Italy; 47 Ospedale dei bambini di Brescia - Spedali civili di Brescia, Brescia, Italy; 48 UOC Pediatria ASST Lariana, San Fermo della Battaglia, Italy; 49 ASST Cremona, Cremona, Italy; 50 Ospedale Manzoni di Lecco, Lecco, Italy; 51 Ospedale Maggiore di Lodi, Lodi, Italy; 52 Ospedale San Gerardo, Monza, Italy; 53 Ospedale Civile di Vimercate, Vimercate, Italy; 54 Presidio Ospedaliero di Legnano – ASST OVEST MI, Legnano, Italy; 55 Ospedale di Busto Arsizio - ASST Valle Olona, Busto Arsizio, Italy; 56 ASST Ovest Milanese - Presidio Ospedaliero Magenta, Magenta, Italy; 57 Ospedale Predabissi - ASST Melegnano e della Martesana, Vizzolo Predabissi, Italy; 58 Ospedale S. Maria delle Stelle, Melzo, Italy; 59 Ospedale dei bambini Vittore Buzzi, Milan, Italy; 60 IRCCS Ospedale San Raffaele, Milan, Italy; 61 Ospedale Macedonio Melloni -Ospedale Fatebenefratelli, Milan, Italy; 62 Grande Ospedale Metropolitano Niguarda, Milan, Italy; 63 Ospedale Fatebenefratelli, Milan, Italy; 64 Azienda Ospedaliera Carlo Poma, Mantua, Italy; 65 Clinica Pediatrica Università di Pavia - Fondazione IRCCS Policlinico San Matteo, Pavia, Italy; 66 Ospedale di Sondrio, Sondrio, Italy; 67 Pronto Soccorso Pediatrico Ospedale F. Del Ponte, Varese, Italy; 68 Ospedale Generale Provinciale di Saronno, Saronno, Italy; 69 Azienda Ospedaliero Universitaria Ospedali Riuniti, Ancona, Italy; 70 Ospedale “C. e G. Mazzoni”, Ascoli Piceno, Italy; 71 Ospedale Augusto Murri, Fermo, Italy; 72 Ospedale di Macerata, Macerata, Italy; 73 Ospedale Santa Maria della Misericordia di Urbino, Pesaro e Urbino, Italy; 74 Ospedale Ferdinando Veneziale, Isernia, Italy; 75 Ospedale Infantile “Cesare Arrigo”, Alessandria, Italy; 76 Ospedale degli Infermi, Biella, Italy; 77 Azienda Sanitario Ospedaliera S.Croce e Carle di Cuneo, Cuneo, Italy; 78 Ospedale SS Trinità, Borgomanero, Italy; 79 Ospedale Maggiore della Carità Novara, Novara, Italy; 80 Ospedale Martini, Turin, Italy; 81 Ospedale Infantile Regina Margherita, Turin, Italy; 82 Ospedale Castelli, Verbania, Italy; 83 Ospedale Sant’Andrea, Vercelli, Italy; 84 Ospedale L. Bonomo ASL BAT, Andria, Italy; 85 Ospedale Mons. Dimiccoli, Barletta, Italy; 86 Ospedale Giovanni XXIII, Bari, Italy; 87 Presidio di Brindisi “Di Summa - Perrino”, Brindisi, Italy; 88 Presidio Ospedaliero Scorrano, Scorrano, Italy; 89 Ospedali Riuniti, Foggia, Italy; 90 Presidio Ospedaliero Centrale - SS. Annunziata, Taranto, Italy; 91 Ospedale San Michele - Azienda Ospedaliera Brotzu, Cagliari, Italy; 92 C.T.O., Iglesias, Italy; 93 Presidio Ospedaliero San Francesco, Nuoro, Italy; 94 Ospedale Nostra Signora della Mercede di Lanusei, Ogliastra, Italy; 95 Ospedale San Martino di Oristano, Oristano, Italy; 96 Ospedale Paolo Dettori, Tempio Pausania, Italy; 97 Azienda Ospedaliera Universitaria di Sassari, Sassari, Italy; 98 Ospedale San Giovanni di Dio, Agrigento, Italy; 99 Presidio Ospedaliero “S.Elia”, Caltanissetta, Italy; 100 Azienda Ospedaliera Universitaria Gaetano Martino, Messina, Italy; 101 Ospedale Pediatrico “G. Di Cristina” - Arnas Civico, Palermo, Italy; 102 Presidio Ospedaliero Giovanni Paolo II, Ragusa, Italy; 103 Presidio Ospedaliero S. Antonio Abate, Trapani, Italy; 104 Ospedale Umberto I di Siracusa - Ospedale A. Rizza, Siracusa, Italy; 105 Ospedale di Avola H. G. di Maria, Avola, Italy; 106 Ospedale di Grosseto Misericordia, Grosseto, Italy; 107 Ospedale Santa Chiara, AOUP, Pisa, Italy; 108 Azienda Ospedaliera-Universitaria Anna Meyer, Florence, Italy; 109 Ospedali Riuniti Livorno, Livorno, Italy; 110 Ospedale San Luca, Lucca, Italy; 111 Nuovo Ospedale Prato-Santo Stefano, Prato, Italy; 112 Ospedale di Bolzano, Bolzano, Italy; 113 Ospedale S. Chiara di Trento, Trento, Italy; 114 Nuovo Ospedale S. Giovanni Battista, Foligno, Italy; 115 Ospedale S. Maria della Misericordia, Perugia, Italy; 116 Ospedale Beauregard, Aosta, Italy; 117 Ospedale San Martino, Belluno, Italy; 118 Azienda Ospedaliera di Padova Dipartimento A.I. per la Salute della Donna e del Bambino, Padua, Italy; 119 UOC di Pediatria, AULSS 5, Rovigo, Italy; 120 Ospedale Ca’ Foncello, Treviso, Italy; 121 Ospedale SS Giovanni e Paolo, Venezia, Italy; 122 Ospedale “G.Fracastoro” - San Bonifacio, Verona, Italy; 123 Ospedale Donna e Bambino Azienda Ospedaliera Universitaria Integrata Verona, Verona, Italy; 124 Ospedale San Bortolo, Vicenza, Italy

**Keywords:** Infants, Urine, Urinary tract, Infection, Guidelines, Survey, Catheter, Emergency department

## Abstract

Urinary tract infections (UTIs) are among the most frequent bacterial diseases in infants and children. Physician adherence to recommendations is notoriously often poor, but no data are available on UTIs management in the emergency setting. In this multicenter national study, we investigated the policies regarding UTIs management in children aged 2 months to 3 years in Italian emergency units. Between April and June 2021, directors of the emergency units were invited to answer an online survey on the following items: diagnostic approach to children with fever without an apparent source, therapeutic approach to UTIs, the use of kidney and urinary tract ultrasound, and the criteria for hospitalization. A total of 121 (89%) out of 139 of invited units participated in the study. Overall, units manage children with a suspected or confirmed UTI according to available recommendations for most of the items. However, in almost 80% (*n* = 94) of units, a sterile perineal bag is used to collect urine for culture. When urine is collected by cathether, heterogeneity exists on the threshold of bacterial load considered for UTI diagnosis.

*Conclusions:* Available recommendations on UTIs in children are followed by Italian emergency units for most of the items. However, the methods to collect urine specimens for culture, one of the crucial steps of the diagnostic work-up, often do not align with current recommendations and CFU thresholds considered for diagnosis largely vary among centers. Efforts should be addressed to validate and implement new child and family friendly urine collection techniques.

**Table:** 

**What is Known:** *• Several guidelines are published on the management of children with suspected or confirmed urinary tract infection.* • *No data are available on the management of pediatric urinary tract infections in the emergency setting.*	
**What is New:** *• Almost 80% of the Italian emergency units employ a sterile perineal bag to collect urine for culture.* *• Diagnostic CFU thresholds largely vary among centers.*

**Supplementary Information:**

The online version contains supplementary material available at 10.1007/s00431-022-04457-0.

## Introduction

Urinary tract infections (UTIs) are among the most common bacterial diseases in childhood. To improve their management, both national and international guidelines have been developed in the last decades [1–8]. However, physician adherence to available recommendations is often poor [9]. For instance, previous surveys among primary care physicians showed that antimicrobials are widely prescribed for outpatient UTI cases without urine culture testing [10, 11]. The difficultness associated to the urine collection might, at least in part, explain these findings [12, 13].

Although UTIs are a frequent cause of emergency unit encounters [14], no data are available on their management in this setting. Therefore, the aim of this study was to investigate the policies regarding pediatric UTIs management in emergency units.

## Material and methods

We conducted a multicenter national survey (the Italian Urinary Tract Infection—ItaUTI—study) among Italian emergency units between April and June 2021. Eligible for this study were all (both general and pediatric) emergency units taking care of children.

### Invitation of the units 

The heads of the emergency units were invited to fill-in an online structured survey on Google Forms platform. To obtain representative, national data, all directors of the emergency units of the national society of pediatric emergency physicians (“Accademia Medica Infermieristica di Emergenza e Terapia Intensiva Pediatrica, AMIETIP”) were invited. Moreover, we invited the directors of the main emergency units of Italian provinces even if not affiliated to the AMIETIP society. Three e-mail reminders (one every two weeks) were sent. If no answer was obtained after these attempts, a phone call to the unit was performed.

### Questionnaire development

The survey was initially developed by two pediatric nephrologists (G.M. and M.G.B.) and two pediatric emergency physicians (G.P.M. and F.C.) based on current Italian recommendations on UTIs management [2] on subjects aged 2 months to 3 years. A pilot test was then undertaken among 10 members or active collaborators of the scientific committee of AMIETIP. Two of them asked to modify only one question to increase its clarity (overall agreement of 95%). The final version of the questionnaire (supplementary material, section “Methods”) comprised three main sections: (1) the first section addressed the characteristics of the emergency unit including the name and town of the hospital, the total number of children and the number of children with UTIs seen on average every year, the frequency of pediatric infectious disease or kidney disease specialist consultations, and the availability of written recommendations on UTIs diagnosis and management in the emergency unit; (2) the second section investigated the diagnostic approach to children with fever without an apparent source including the use of urine dipstick in children, the urine dipstick findings considered for the diagnosis of UTI, the urine microscopy employment, the methods of urine collection for urinary dipstick and urine culture, the threshold of colony-forming units (CFU)/ml assumed for the diagnosis of UTI on urine culture, and the blood testing (e.g. blood cell count, inflammatory markers, renal function and electrolytes) in children with a suspected UTI; (3) the last section addressed policies about therapeutic approach of confirmed cases, including timing of antimicrobial prescription, choice of empiric antimicrobial treatment, the use of kidney-urinary tract ultrasound and the criteria for hospitalization. Only a single choice was possible for all questions, except for the question concerning the criteria for hospitalization for which more than one option could be selected. The answers were automatically collected into an online database and then transferred to an excel spreadsheet.

### Data analysis

No missing data were expected since completion of all answers was mandatory. Categorical variables were reported as frequencies and percentages. The percentages of agreement between the policies adopted by the emergency units and recommendations from the Italian [2] and European Urology Association [1], the National Institute for Health and Care Excellence (NICE) [5], and the American Academy of Pediatrics [7, 8] guidelines were also assessed. For questions with a discrepancy > 50% between the units’ answers and the Italian guidelines recommendations, the Fisher exact test was used to assess a possible difference between units managing ≥ 100 UTIs cases per year and the other units and units with available written recommendations on UTIs diagnosis and management and the other units. A p < 0.05 was assumed as significant.

## Results

### Participant unit characteristics

**Fig. 1:**
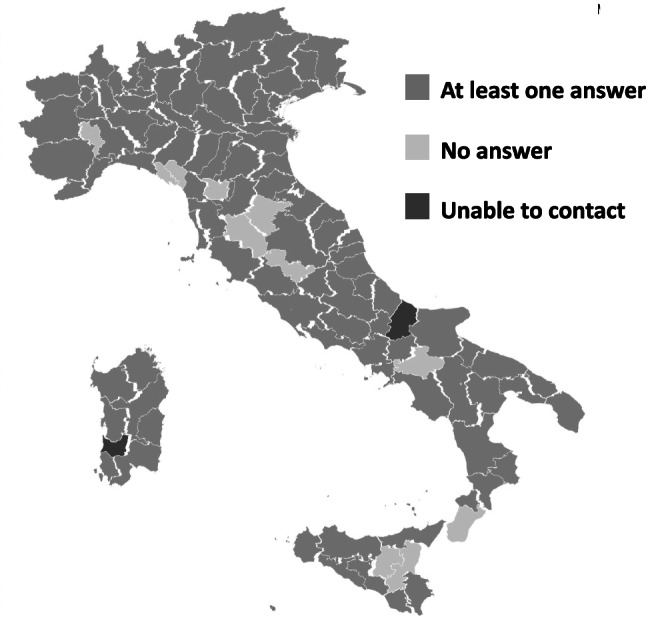
Italian provinces with at least one unit invited: in grey color those with at least one participant center, in light gray color those without any participating unit. We were not able to invite any unit in two provinces (dark gray color)

A total of 121 (89%) out of 139 of invited units participated in the survey. The characteristics of the participating units are given in Table 1. The centers were located throughout Italy as shown in the Fig. 1.

**Table 1:** Characteristics of the Italian emergency units involved in the survey

	***N* (%)**
**Geographical area**	
North	62 (51)
Center	25 (21)
South and Islands	34 (28)
**Number of visits per year**	
< 5000	30 (25)
5000–10,000	33 (27)
10,000–20,000	35 (29)
> 20,000	23 (19)
**Number of pediatric UTI managed per year**	
< 50	46 (38)
50–100	45 (37)
100–200	14 (12)
> 200	16 (13)
**Frequency of consultation with a pediatric infectious disease specialist for UTI management**	
Never	109 (90)
< 50%	0 (0.0)
≥ 50%	0 (0.0)
Always	12 (10)
**Frequency of consultation with a pediatric nephrologist for UTI management**	
Never	75 (62)
< 50%	33 (27)
≥ 50%	9 (7.4)
Always	4 (3.3)
**Availability of recommendations on UTI management**	
Yes	90 (74)
No	31 (26)

### Diagnostic approach to pediatric UTIs

#### Urinary dipstick

In the majority (*N* = 102, 84%) of units, a urine dipstick is always performed in the diagnostic work-up of a febrile child with no apparent source. In 14% of the units, a urinary dipstick is performed in ≥ 50% of cases, while in 2 units in < 50% of cases. For this purpose, a sterile perineal bag is employed in 95% (*N* = 115) of the units and in only 4 (3.3%), a clean catch is used. The concurrent positivity for leukocyte esterase and nitrites is considered for the diagnosis of UTIs in 76% of units and the positivity for only leukocyte esterase in 13%.

#### Urine culture

In more than three quarter of units (*n* = 94, 78%), a sterile perineal bag is used to collect urine for culture. In only 16 (13%) and 9 (11%) of the units, either a bladder catheter or a clean catch of urine is chosen, respectively.

**Fig. 2:**
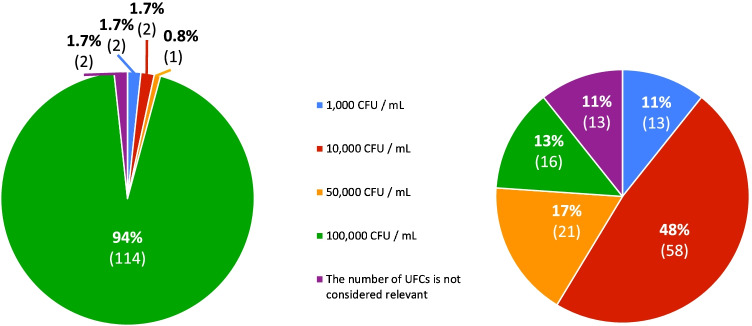
Left: bacterial load (colony-forming units/ml) considered diagnostic for urinary tract infection in urine collected by perineal bag. Right: bacterial load (colony-forming units/ml) considered diagnostic in urine collected by bladder catheter

The CFU/ml thresholds adopted for the diagnosis of UTI are shown in Fig. 2: overall, 94% of centers consider 100.000 CFU/ml in specimens collected by sterile perineal bag and 50% consider 10.000 CFU/ml in specimens collected by bladder catheter.

#### Further investigations

Microscopic urinalysis is never performed in 33 (27%) units. On the contrary, in 30 (25%) units, it is always requested in the diagnostic work-up of UTIs. Blood tests are always performed in 45% (*N* = 56) of the units and never in only 7% (*N* = 8).

### Therapeutic approach and management of confirmed cases of UTIs

#### Antimicrobial therapy

An antimicrobial treatment is prescribed immediately after urine collection for culture in the majority (*N* = 105, 87%) of units. On the contrary, antimicrobial treatment is immediately prescribed without urine collection for bacteriological studies in a low percentage (*N* = 4, 3.3%) of units. Amoxicillin-Clavulanic acid is prescribed as first-line empiric treatment (*N* = 105, 87%) at a dosage between 50 and 90 mg/kg body weight (*N* = 100, 95%) in most units. Amoxicillin alone is used by a minority of units (*N* = 10, 8.3%).

#### Criteria for hospitalization

The criteria adopted for hospitalization are given in Table 2. The persistence of fever despite a 3-day course of antimicrobial treatment and an expected poor adherence to treatment are the most common criteria implemented in most units (83% and 78%, respectively).

**Table 2:** Criteria for hospitalization of children with UTIs. The question allowed multiple answers

	***N (%)***
Persistence of fever despite an appropriate 3-day course of antimicrobial treatment	101 (84)
Expected poor adherence to treatment	94 (78)
Age < 6 months	72 (59)
Fever with chills	59 (48)
Children on antibiotic prophylaxis for previous UTIs episodes	54 (45)
Age < 12 months	22 (18)

#### Kidney and urinary tract ultrasound

**Fig. 3:**
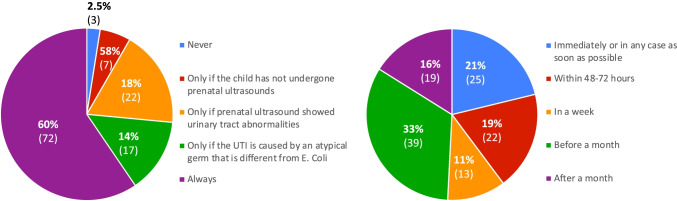
Left: indications for kidney-urinary tract ultrasound in a febrile child in a good condition with a first episode of UTI. Right: timing of kidney-urinary tract ultrasound

Most units (*N* = 72, 60%) always perform a kidney and urinary tract ultrasound following the first episode of UTI. A total of 22 units (18%) perform an ultrasound only in children with pathological prenatal ultrasound findings and 17 (14%) if UTI is caused by microorganism other than *E. coli*. Approximately 40% (*N* = 47) of units perform ultrasound immediately or within 48–72 h and 19 (16%) one month after the UTI episode (Fig. 3). The remaining results are reported in the online supplementary material.

The agreement between the policy adopted by the emergency units and the recommendations of the Italian and European Urology Association, the NICE, and the American Academy of Pediatrics guidelines and are given in Table 3. The features considered in the urinary dipstick for the diagnosis of an upper urinary tract infection in a febrile child, the method used for urine collection for culture, the bacterial load considered diagnostic for a UTI in urine, the request of blood exam and the timing of kidney and urinary tract ultrasound were the items with a discrepancy > 50% between the Italian guidelines and units’ answers. Units managing ≥ 100 UTIs yearly used more frequently bladder catheterization to collect urine compared to the other units (27% vs 8.8%, *p* = 0.02). No other difference was observed for units managing ≥ 100 UTIs or those with the availability of written recommendations on the other items.

**Table 3:** Agreement between available recommendations on UTIs for children and the policies of the emergency units (*N* = 121) included in the survey

**Item**	**Italian guidelines**	**AAP guidelines**	**NICE guidelines**	**European Society of Urology guidelines**
Collection of a urinary dipstick in subjects with fever without an apparent source of infection	84%	NA	84%	84%
Urine collection for urinary dipstick	98%	1.6%	98%	98%
Features considered in the urinary dipstick for the diagnosis of an upper urinary tract infection in a febrile child	13%	13%	75%	75%
Urine collection for culture	13%	13%	9.1%	13%
Bacterial load considered diagnostic for a UTI in urine collected by perineal sterile bag	94%	NA	NA	NA
Bacterial load (colony-forming units / CFU) considered diagnostic for a UTI in urine collected by bladder catheter	48%	17%	11%	59%
Blood exams	24%	24%	24%	NA
Empirical antimicrobial oral treatment	87%	87%	87%	4.9%
Dosage of oral Amoxicillin Clavulanate	88%	0.8%	88%	NA
Timing of antimicrobial oral treatment	87%	NA	87%	87%
Kidney and urinary tract ultrasound at the first UTI episode	60%	60%	14%^a^	60%
Timing of kidney and urinary tract ultrasound	33%	49%	NA	21%

*NA* not applicable, *AAP* American Academy of Pediatrics, *NICE* National Institute for Health and Care Excellence

^a^ Kidney and urinary tract ultrasound is also recommended in cases > 6 months of age

## Discussion

Here, we report the first study investigating the diagnosis and management of pediatric UTIs in the emergency units. The findings of this nationwide survey, which had a remarkably good response rate at almost 90%, point out that children aged 2 months to 3 years with a suspected or confirmed UTI are mostly managed according to currently available recommendations.

On the other hand, the survey shows that the adopted techniques to collect urine for culture are often not performed in accordance with the guidelines, and CFU thresholds for diagnosis vary among centers.

The discussion will focus on three points of the survey: 1. the diagnostic work-up, 2. the criteria for hospitalization, 3. the therapeutic approach.

In febrile infants without an apparent source of fever, urinalysis is currently recommended by many authorities [1–3]. In line with this advice, our study shows that in most of the Italian emergency units (84%), a urine dipstick is obtained in children without an apparent source of fever. However, some units do not routinely perform a urine dipstick in these patients. The latter finding should be considered since the rule-out of a UTI may be helpful to establish the likelihood of a viral infection and avoid an overprescription of antimicrobials.

The evidence of pyuria is currently considered more relevant than the presence of nitrituria in the diagnostic workup of UTIs [2]. Most units require the positivity for both pyuria and nitrituria to rule in a diagnosis of UTIs, while a minority of units considers leucocytes only. This attitude likely reflects that currently available dipsticks determine both parameters. Considering the tendency to overprescribe antimicrobials [15], future studies should investigate the impact of these different attitudes on the management of suspected cases of UTI.

The growth of pathogens in an uncontaminated urine specimen is the cornerstone for UTI diagnosis [16]. For this purpose, bladder catheterization and suprapubic puncture are considered the gold standard to collect urine both in infants and in not-toiled trained children [2]. Due to the risk of contamination, the current guidelines advise against the use of perineal bag [1–8]. This survey shows that sterile perineal bag is used in most of the emergency units. This finding is in line with figures observed among European primary care physicians [10]. It is fairly predictable that invasive techniques such as bladder catheterization and suprapubic puncture might be challenging in the outpatients setting. However, the widespread use of perineal bag also in the emergency units suggests that this attitude is likely an ingrained habit among pediatricians. Emergency care providers would rather rarely employ bladder catheterization or suprapubic puncture likely because these are personnel and time intensive, and a possible source of pain for children and distress for caregivers [17, 18]. New strategies, such as the collection of urine from nappy pad or bladder stimulation technique to obtain midstream urine, might be an interesting and promising alternative [16, 19]. We suggest that the emergency units review their practice by considering and validating new urine collection methods for culture that may be effective and safe also in centers with limited staffing and resources, and in small centers.

As for the CFU cut-off for UTI diagnosis, the standard threshold of ≥ 10^5^ CFU/ml on urine obtained in a sterile perineal bag is utilized by almost all units. The threshold for urine collected by more invasive procedures is heterogenous. These data confirm that no consensus exists as to what threshold should be considered for the diagnosis of UTIs [20] and new studies are needed to define this gap of knowledge.

More and more data suggest that children affected by a febrile UTI can be feasibly managed as outpatients [21]. The results of our survey are in line with this approach, because only an expected poor adherence to treatment and fever persistence despite an appropriate 3-day antimicrobial course are reasons for hospitalization in most units.

The results of this study point out that oral Amoxicillin, alone or more frequently associated with Clavulanate, is the empirical treatment prescribed by most emergency units. This policy likely reflects the fact that in Italy, four out of five community acquired childhood UTIs are caused by *E. coli* and ~ 70% of these pathogens are sensitive to Amoxicilline-Clavalunate [22].

This study has limitations and strengths. First, our questionnaire was pilot tested but not validated. Second, the responses are based on self-reports and might not fully reflect the day-to-day clinical practice. Future studies with data from medical records of the patients might further support the results of this study and quantitate the magnitude of overtreatment associated with the use of perineal bag instead of that of bladder catheter for urine culture. Third, the study was limited to diagnosis and management of UTIs within Italian emergency units and not all the Italian units and scientific societies were invited. Finally, we did not investigate if units followed Italian or other guidelines on UTI. The major strength is that this nationwide inquiry benefited from a response rate of about 90%. Information was gathered from units throughout all Italy including both small and large emergency units. Finally, almost all aspects of UTI management in children aged 2 months to 3 years in the acute setting were investigated.

In conclusion, this study shows that currently available recommendations on UTIs in children are followed by Italian emergency units for most of the items. On the other hand, the methods to collect urine specimens for culture, one of the crucial steps for UTI diagnosis, are not performed according to the recommendations and CFU thresholds are heterogenous. Efforts should be addressed to develop and validate new child and family friendly urine collection techniques.

## Supplementary information

Below is the link to the electronic supplementary material.

Supplementary file1 (PDF 378 KB)

## Data Availability

Upon reasonable request to the corresponding author.

## Abbreviations

AAP: American Academy of Pediatrics
NA: Not applicable
NICE: National Institute for Health and Care Excellence
UTI: Urinary tract infection
