# Correction: Diagnosis and management of urinary tract infections in children aged 2 months to 3 years in the Italian emergency units: the ItaUTI study

**DOI:** 10.1007/s00431-022-04479-8

**Published:** 2022-04-28

**Authors:** Francesca Cenzato, Gregorio P. Milani, Angela Amigoni, Francesca Sperotto, Mario G. Bianchetti, Carlo Agostoni, Giovanni Montini, on behalf of the ItaUTI study group, Giovanni Farello, Francesco Chiarelli, Rita Greco, Franco Di Lollo, Fabio Rocco Forte, Sergio Manieri, Luigi Carpino, Mimma Caloiero, Anastasia Cirisano, Salvatore Braghò, Roberto Della Casa, Felice Nunziata, Carmine Pecoraro, Rosario Pacifico, Marcello Lanari, Chiara Ghizzi, Laura Serra, Marcello Stella, Giuseppe Maggiore, Roberto Fiorini, Icilio Dodi, Andrea Morelli, Lorenzo Lughetti, Andrea Cella, Gianluca Vergine, Alessandro De Fanti, Danica Dragovic, Daniele Santori, Giorgio Cozzi, Paola Cogo, Marilena Raponi, Riccardo Lubrano, Mauro de Martinis, Antonio Gatto, Maria Antonietta Barbieri, Antonino Reale, Giorgio Bracaglia, Emanuela Piccotti, Riccardo Borea, Alberto Gaiero, Laura Martelli, Alberto Arrighini, Paola Cianci, Claudio Cavalli, Leonardina De Santis, Benedetta Chiara Pietra, Andrea Biondi, Marco Sala, Laura M. Pogliani, Simonetta Cherubini, Marta Bellini, Paola Bruni, Giovanni Traina, Paola Tommasi, Paolo Del Barba, Sergio Arrigoni, Filippo M. Salvini, Luca Bernardo, Giuseppe Bertolozzi, Silvia Fasoli, Gian Luigi Marseglia, Emilio Palumbo, Annalisa Bosco, Gianpaolo Mirri, Elisabetta Fabiani, Ermanno Ruffini, Luisa Pieragostini, Martina Fornaro, Gabriele Ripanti, Donnina Pannoni, Felici Enrico, Anna Perona, Eleonora Tappi, Oscar Nis Haitink, Ivana Rabbone, Pina Teresa Capalbo, Antonio Urbino, Andrea Guala, Gianluca Cosi, Maria Gabriella Barracchia, Baldassarre Martire, Fabio Cardinale, Fulvio Moramarco, Carmelo Perrone, Angelo Campanozzi, Valerio Cecinati, Alessandro Canetto, Ciro Clemente, Antonio Cualbu, Fabio Narducci, Giuseppina Mula, Pasquale Bulciolu, Roberto Antonucci, Giuseppe Gramaglia, Giuseppe Cavaleri, Carmelo Salpietro, Giovanni Corsello, Rosario Salvo, Marcello Palmeri, Maria Assunta Vitale, Ambra Morgano, Susanna Falorni, Diego Peroni, Stefano Masi, Alessio Bertini, Angelina Vaccaro, Pierluigi Vasarri, Petra Reinstadler, Massimo Soffiati, Maurizio Stefanelli, Alberto Verrotti di Pianella, Catherine Bertone, Stefano Marzini, Liviana Da Dalt, Simone Rugolotto, Floriana Scozzola, Luca Ecclesio Livio, Mauro Cinquetti, Davide Silvagni, Massimo Bellettato

**Affiliations:** 1 Pediatric Emergency Department, Fondazione IRCCS Ca’ Granda Ospedale Maggiore Policlinico, Milan, Italy; 2 Department of Clinical Sciences and Community Health, Università Degli Studi Di Milano, Milan, Italy; 3 Pediatric Intensive Care Unit, Department of Women’s and Children’s Health, University Hospital of Padua, Padua, Italy; 4 Family Medicine Institute, Faculty of Biomedical Sciences, Università Della Svizzera Italiana, Lugano, Switzerland; 5 Pediatric Nephrology, Dialysis and Transplant Unit, Fondazione IRCCS Ca’ Granda Ospedale Maggiore Policlinico, Milan, Italy; 6 Presidio Ospedaliero “San Salvatore” - Clinica Pediatrica Università dell’Aquila, L’Aquila, Italy; 7 Department of Paediatrics, University of Chieti, Chieti, Italy; 8 Presidio Ospedaliero “S. Spirito” Pescara - UOC Pediatria di Pescara, Pescara, Italy; 9 Ospedale “Giuseppe Mazzini”, Teramo, Italy; 10 Presidio Ospedaliero “Madonna delle Grazie”, Matera, Italy; 11 Ospedale San Carlo, Potenza, Italy; 12 Ospedale Annunziata, Cosenza, Italy; 13 Ospedale Giovanni Paolo II, Lamezia Terme, Italy; 14 Ospedale S. Giovanni di Dio, Crotone, Italy; 15 Presidio Ospedaliero “G. Jazzolino”, Vibo Valentia, Italy; 16 Azienda Ospedaliera San Pio, Benevento, Italy; 17 Ospedale Sant’Anna e San Sebastiano, Caserta, Italy; 18 Azienda Ospedaliera Pediatrica Santobono-Pausilipon, Naples, Italy; 19 Azienda Ospedaliera Universitaria S.Giovanni e Ruggi, Salerno, Italy; 20 IRCCS-Policlinico universitario di Sant’Orsola, Bologna, Italy; 21 Ospedale Maggiore Bologna, Bologna, Italy; 22 Ospedale Santa Maria della Scaletta, Imola, Italy; 23 Ospedale Bufalini-Marconi, Cesena, Italy; 24 Ospedale Sant’Anna, Ferrara, Italy; 25 Ospedale di Fidenza, Fidenza, Italy; 26 Ospedale dei bambini “Pietro Barilla” - Azienda Ospedaliero Universitaria di Parma, Parma, Italy; 27 Ospedale Santa Maria delle Croci, Ravenna, Italy; 28 Policlinico di Modena, Modena, Italy; 29 Ospedale Guglielmo da Saliceto, Piacenza, Italy; 30 Ospedale degli Infermi, Rimini, Italy; 31 Arcispedale Santa Maria Nuova, Reggio Emilia, Italy; 32 Ospedale San Polo, Monfalcone, Italy; 33 Ospedale Santa Maria degli Angeli, Pordenone, Italy; 34 IRCCS Materno Infantile Burlo Garofolo, Trieste, Italy; 35 Ospedale Santa Maria della Misericordia, Udine, Italy; 36 Presidio Ospedaliero di Cassino, Frosinone, Italy; 37 Ospedale Santa Maria Goretti, Latina, Italy; 38 Ospedale San Camillo de Lellis, Rieti, Italy; 39 Policlinico Universitario Agostino Gemelli, Rome, Italy; 40 Ospedale Bambino Gesù Palidoro, Rome, Italy; 41 Ospedale Pediatrico Bambin Gesù, Rome, Italy; 42 Ospedale Belcolle, Viterbo, Italy; 43 IRCCS G. Gaslini, Genua, Italy; 44 Ospedale Civile di Imperia, Imperia, Italy; 45 Ospedale San Paolo / Santa Corona Pietra Ligure, Savona, Italy; 46 Ospedale Papa Giovanni XXIII, Bergamo, Italy; 47 Ospedale dei bambini di Brescia - Spedali civili di Brescia, Brescia, Italy; 48 UOC Pediatria ASST Lariana, San Fermo della Battaglia, Italy; 49 ASST Cremona, Cremona, Italy; 50 Ospedale Manzoni di Lecco, Lecco, Italy; 51 Ospedale Maggiore di Lodi, Lodi, Italy; 52 Ospedale San Gerardo, Monza, Italy; 53 Ospedale Civile di Vimercate, Vimercate, Italy; 54 Presidio Ospedaliero di Legnano – ASST OVEST MI, Legnano, Italy; 55 Ospedale di Busto Arsizio - ASST Valle Olona, Busto Arsizio, Italy; 56 ASST Ovest Milanese - Presidio Ospedaliero Magenta, Magenta, Italy; 57 Ospedale Predabissi - ASST Melegnano e della Martesana, Vizzolo Predabissi, Italy; 58 Ospedale S. Maria delle Stelle, Melzo, Italy; 59 Ospedale dei bambini Vittore Buzzi, Milan, Italy; 60 IRCCS Ospedale San Raffaele, Milan, Italy; 61 Ospedale Macedonio Melloni -Ospedale Fatebenefratelli, Milan, Italy; 62 Grande Ospedale Metropolitano Niguarda, Milan, Italy; 63 Ospedale Fatebenefratelli, Milan, Italy; 64 Azienda Ospedaliera Carlo Poma, Mantua, Italy; 65 Clinica Pediatrica Università di Pavia - Fondazione IRCCS Policlinico San Matteo, Pavia, Italy; 66 Ospedale di Sondrio, Sondrio, Italy; 67 Pronto Soccorso Pediatrico Ospedale F. Del Ponte, Varese, Italy; 68 Ospedale Generale Provinciale di Saronno, Saronno, Italy; 69 Azienda Ospedaliero Universitaria Ospedali Riuniti, Ancona, Italy; 70 Ospedale “C. e G. Mazzoni”, Ascoli Piceno, Italy; 71 Ospedale Augusto Murri, Fermo, Italy; 72 Ospedale di Macerata, Macerata, Italy; 73 Ospedale Santa Maria della Misericordia di Urbino, Pesaro e Urbino, Italy; 74 Ospedale Ferdinando Veneziale, Isernia, Italy; 75 Ospedale Infantile “Cesare Arrigo”, Alessandria, Italy; 76 Ospedale degli Infermi, Biella, Italy; 77 Azienda Sanitario Ospedaliera S.Croce e Carle di Cuneo, Cuneo, Italy; 78 Ospedale SS Trinità, Borgomanero, Italy; 79 Ospedale Maggiore della Carità Novara, Novara, Italy; 80 Ospedale Martini, Turin, Italy; 81 Ospedale Infantile Regina Margherita, Turin, Italy; 82 Ospedale Castelli, Verbania, Italy; 83 Ospedale Sant’Andrea, Vercelli, Italy; 84 Ospedale L. Bonomo ASL BAT, Andria, Italy; 85 Ospedale Mons. Dimiccoli, Barletta, Italy; 86 Ospedale Giovanni XXIII, Bari, Italy; 87 Presidio di Brindisi “Di Summa - Perrino”, Brindisi, Italy; 88 Presidio Ospedaliero Scorrano, Scorrano, Italy; 89 Ospedali Riuniti, Foggia, Italy; 90 Presidio Ospedaliero Centrale - SS. Annunziata, Taranto, Italy; 91 Ospedale San Michele - Azienda Ospedaliera Brotzu, Cagliari, Italy; 92 C.T.O., Iglesias, Italy; 93 Presidio Ospedaliero San Francesco, Nuoro, Italy; 94 Ospedale Nostra Signora della Mercede di Lanusei, Ogliastra, Italy; 95 Ospedale San Martino di Oristano, Oristano, Italy; 96 Ospedale Paolo Dettori, Tempio Pausania, Italy; 97 Azienda Ospedaliera Universitaria di Sassari, Sassari, Italy; 98 Ospedale San Giovanni di Dio, Agrigento, Italy; 99 Presidio Ospedaliero “S.Elia”, Caltanissetta, Italy; 100 Azienda Ospedaliera Universitaria Gaetano Martino, Messina, Italy; 101 Ospedale Pediatrico “G. Di Cristina” - Arnas Civico, Palermo, Italy; 102 Presidio Ospedaliero Giovanni Paolo II, Ragusa, Italy; 103 Presidio Ospedaliero S. Antonio Abate, Trapani, Italy; 104 Ospedale Umberto I di Siracusa - Ospedale A. Rizza, Siracusa, Italy; 105 Ospedale di Avola H. G. di Maria, Avola, Italy; 106 Ospedale di Grosseto Misericordia, Grosseto, Italy; 107 Ospedale Santa Chiara, AOUP, Pisa, Italy; 108 Azienda Ospedaliera-Universitaria Anna Meyer, Florence, Italy; 109 Ospedali Riuniti Livorno, Livorno, Italy; 110 Ospedale San Luca, Lucca, Italy; 111 Nuovo Ospedale Prato-Santo Stefano, Prato, Italy; 112 Ospedale di Bolzano, Bolzano, Italy; 113 Ospedale S. Chiara di Trento, Trento, Italy; 114 Nuovo Ospedale S. Giovanni Battista, Foligno, Italy; 115 Ospedale S. Maria della Misericordia, Perugia, Italy; 116 Ospedale Beauregard, Aosta, Italy; 117 Ospedale San Martino, Belluno, Italy; 118 Azienda Ospedaliera di Padova Dipartimento A.I. per la Salute della Donna e del Bambino, Padua, Italy; 119 UOC di Pediatria, AULSS 5, Rovigo, Italy; 120 Ospedale Ca’ Foncello, Treviso, Italy; 121 Ospedale SS Giovanni e Paolo, Venezia, Italy; 122 Ospedale “G.Fracastoro” - San Bonifacio, Verona, Italy; 123 Ospedale Donna e Bambino Azienda Ospedaliera Universitaria Integrata Verona, Verona, Italy; 124 Ospedale San Bortolo, Vicenza, Italy

## Correction to: European Journal of Pediatrics https://doi.org/10.1007/s00431-022-04457-0

In the original published version of this article, the Collaborators’ names were not presented in the authorship section. The names correctly presented above.

The original article has been corrected.

