# Supplementary material for: Diagnosis and management of urinary tract infections in children aged 2 months to 3 years in the Italian emergency units: the ItaUTI study
Source: Eur J Pediatr. 2022 Apr 6;181(7):2663–71. doi: 10.1007/s00431-022-04457-0 (PMC9192484; doi:10.1007/s00431-022-04457-0)
Supplement: Supplementary file 1 — Supplementary file1 (PDF 378 KB) [file 431_2022_4457_MOESM1_ESM.pdf]

# Supplementary material

# Management of urinary tract infections in children 2 months to 3 years of age in the emergency room

## Questionnaires employed for the survey and results

**1)** Hospital name and city

**Result**

North Italy

| Name of the Hospital | City |
|---|---|
| IRCCS-Policlinico universitario di Sant'Orsola | Bologna |
| Ospedale Santa Maria della Scaletta | Imola |
| Ospedale Maggiore Bologna | Bologna |
| Ospedale Bufalini-Marconi | Cesena |
| Ospedale Sant'Anna | Ferrara |
| Ospedale di Fidenza | Fidenza |
| Ospedale dei bambini "Pietro Barilla" - Azienda Ospedaliero Universitaria di Parma | Parma |
| Ospedale Santa Maria delle Croci | Ravenna |
| Policlinico di Modena | Modena |
| Ospedale Guglielmo da Saliceto | Piacenza |
| Ospedale degli Infermi | Rimini |
| Arcispedale Santa Maria Nuova | Reggio Emilia |
| Ospedale San Polo | Monfalcone |
| Ospedale Santa Maria degli Angeli | Pordenone |
| IRCCS Materno Infantile Burlo Garofolo | Trieste |
| Ospedale Santa Maria della Misericordia | Udine |
| IRCCS G.Gaslini | Genova |
| Ospedale Civile di Imperia | Imperia |
| Ospedale San Paolo/ Santa Corona Pietra Ligure | Savona |
| Ospedale Papa Giovanni XXIII | Bergamo |
| Ospedale dei bambini di Brescia - Spedali civili di Brescia | Brescia |
| UOC Pediatria ASST Lariana | San Fermo della Battaglia |
| ASST Cremona | Cremona |
| Ospedale Manzoni di Lecco | Lecco |
| Ospedale Maggiore di Lodi | Lodi |
| Ospedale San Gerardo | Monza |
| Ospedale Civile di Vimercate | Vimercate |
| Ospedale di Busto Arsizio - ASST Valle Olona | Busto Arsizio |
| Presidio Ospedaliero di Legnano – ASST OVEST MI | Legnano |
| ASST Ovest Milanese - Presidio Ospedaliero Magenta | Magenta |
| Ospedale Predabissi - ASST Melegnano e della Martesana | Vizzolo Predabissi |
| Ospedale Santa Maria delle Stelle | Melzo |
| Ospedale dei bambini Vittore Buzzi | Milan |
| IRCCS Ospedale San Raffaele | Milan |
| Ospedale Macedonio Melloni | Milan |
| Grande Ospedale Metropolitano Niguarda | Milan |
| Ospedale Fatebenefratelli | Milan |

| | |
|---|---|
| Clinica De Marchi - Fondazione IRCCS Ca' Granda Ospedale Maggiore Policlinico | Milan |
| Azienda Ospedaliera Carlo Poma | Mantua |
| Clinica Pediatrica Università di Pavia - Fondazione IRCCS Policlinico San Matteo | Pavia |
| Ospedale di Sondrio | Sondrio |
| Ospedale Generale Provinciale di Saronno | Saronno |
| Ospedale Filippo del Ponte | Varese |
| Ospedale Infantile "Cesare Arrigo" | Alessandria |
| Ospedale degli Infermi | Biella |
| Azienda Sanitario Ospedaliera S.Croce e Carle di Cuneo | Cuneo |
| Ospedale SS Trinità | Borgomanero |
| Ospedale Maggiore della Carità Novara | Novara |
| Ospedale Martini | Turin |
| Ospedale Infantile Regina Margherita | Turin |
| Ospedale Castelli | Verbania |
| Ospedale Sant'Andrea | Vercelli |
| Ospedale di Bolzano | Bolzano |
| Ospedale S. Chiara di Trento | Trento |
| Ospedale Beauregard | Aosta |
| Ospedale San Martino | Belluno |
| Azienda Ospedaliera di Padova Dipartimento A.I. per la Salute della Donna e del Bambino | Padua |
| Ospedale Santa Maria Regina degli Angeli | Adria |
| Ospedale Santa Maria della Misericordia | Rovigo |
| Ospedale Ca' Foncello | Treviso |
| Ospedale SS Giovanni e Paolo | Venezia |
| Ospedale San Bortolo | Vicenza |
| Ospedale Fracastoro San Bonifacio | Verona |
| Ospedale Donna e Bambino AOUI Verona | Verona |

Center Italy

| **Name of the Hospital** | **City** |
|---|---|
| Azienda Ospedaliero Universitaria Ospedali Riuniti | Ancona |
| Ospedale “C. e G. Mazzoni" | Ascoli Piceno |
| Ospedale Augusto Murri | Fermo |
| Ospedale di Macerata | Macerata |
| Ospedale Santa Maria della Misericordia di Urbino | Pesaro e Urbino |
| Ospedale di Grosseto Misericordia | Grosseto |
| Ospedale Santa Chiara AOUP | Pisa |
| Azienda Ospedaliera-Universitaria Anna Meyer | Florence |
| Ospedali Riuniti Livorno | Livorno |
| Ospedale San Luca | Lucca |
| Nuovo Ospedale Prato-Santo Stefano | Prato |
| Nuovo Ospedale S. Giovanni Battista | Foligno |
| Ospedale S. Maria della Misericordia | Perugia |

South Italy and Islands

| **Name of the Hospital** | **City** |
|---|---|
| Presidio Ospedaliero "San Salvatore" - Clinica Pediatrica Università dell'Aquila | L'Aquila |
| Department of Paediatrics, University of Chieti | Chieti |
| Presidio Ospedaliero "S. Spirito" Pescara - UOC Pediatria di Pescara | Pescara |
| Ospedale “Giuseppe Mazzini” di Teramo | Teramo |
| Presidio Ospedaliero “Madonna delle Grazie” | Matera |

| | |
|---|---|
| Ospedale San Carlo | Potenza |
| Ospedale Annunziata | Cosenza |
| Ospedale Giovanni Paolo II | Lamezia Terme |
| Ospedale S. Giovanni di Dio | Crotone |
| Presidio Ospedaliero “G. Jazzolino” | Vibo Valentia |
| Azienda Ospedaliera San Pio | Benevento |
| Ospedale Sant’Anna e San Sebastiano | Caserta |
| Azienda Ospedaliera Pediatrica Santobono-Pausilipon | Naples |
| Azienda Ospedaliera Universitaria S.Giovanni e Ruggi | Salerno |
| Presidio Ospedaliero di Cassino | Frosinone |
| Ospedale Santa Maria Goretti | Latina |
| Ospedale San Camillo de Lellis | Rieti |
| Policlinico Universitario Agostino Gemelli | Rome |
| Ospedale Bambino Gesù Palidoro | Rome |
| Ospedale Pediatrico Bambin Gesù | Rome |
| Ospedale Belcolle | Viterbo |
| Ospedale Ferdinando Veneziale | Isernia |
| Ospedale L. Bonomo | Andria |
| Ospedale Mons. Dimiccoli | Barletta |
| Ospedale Giovanni XXIII | Bari |
| Presidio di Brindisi "Di Summa - Perrino" | Brindisi |
| Presidio Ospedaliero Scorrano | Scorrano |
| Ospedali Riuniti | Foggia |
| Presidio Ospedaliero Centrale - SS. Annunziata | Taranto |
| Ospedale San Michele - Azienda Ospedaliera Brotzu | Cagliari |
| C.T.O. | Iglesias |
| Presidio Ospedaliero San Francesco | Nuoro |
| Ospedale Nostra Signora della Mercede di Lanusei | Ogliastra |
| Ospedale San Martino di Oristano | Oristano |
| Ospedale Paolo Dettori | Tempio Pausania |
| Azienda Ospedaliera Universitaria di Sassari | Sassari |
| Ospedale San Giovanni di Dio | Agrigento |
| Presidio Ospedaliero “S.Elia” | Caltanissetta |
| Azienda Ospedaliera Universitaria Gaetano Martino | Messina |
| Ospedale Pediatrico "G. Di Cristina" - Arnas Civico | Palermo |
| Presidio Ospedaliero Giovanni Paolo II | Ragusa |
| Presidio Ospedaliero S. Antonio Abate | Trapani |
| Ospedale Umberto I di Siracusa - Ospedale A. Rizza | Siracusa |
| Ospedale di Avola H. G di Maria | Avola |

**2)** Average number of pediatric visits per year in emergency unit:

- <5,000 cases
- 5,000 - 10,000 cases
- 10,000 - 20,000 cases
- >20,000 cases

**Result**
30 (25%) <5,000 cases; 33 (27%) between 5,000 - 10,000 cases; 35 (29%) between 10,000 - 20,000 cases; 23 (19%) >20,000 cases

**3)** Average number of urinary tract infections managed per year in emergency unit:

- <50 cases
- 50 – 100 cases
- 100 – 200 cases

- 200 cases

**Result**
46 (38%) <50 cases; 45 (37%) between 50 - 100 cases; 14 (22%) between 100 - 200 cases; 16 (13%) >200 cases

**4)** To manage a child in a good general condition with a suspected urinary tract infection, do you consult a pediatric infectious disease physician?

- Never
- <50%
- ≥ 50%
- Always

**Result**
109 (90%) Never; 0 (0.0%) <50%; 0 (0.0) ≥ 50%; 12 (10%) Always

**5)** To manage a child in a good general condition with a suspected urinary tract infection, do you consult a pediatric nephrologist?

- Never
- <50%
- ≥ 50%
- Always

**Result**
75 (62%) Never; 33 (27%) <50%; 9 (7.4) ≥ 50%; 4 (3.3%) Always

**6)** Do internal written recommendations exist in your emergency unit for the management of a febrile child with suspected or known urinary tract infection?

- Yes
- No

**Result**
90 (74%) Yes; 31 (26%) No

**7)** How often is urinary dipstick used in the evaluation of a febrile child with no apparent source?

- Never
- <50%
- ≥ 50%
- Always

**Result**

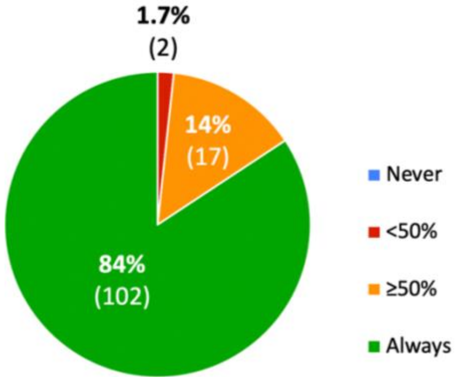

**8)** Which of these tools is most often used to collect urine to perform a urinary dipstick in a child in a good general condition with a suspected clinical picture of urinary tract infection and without sphincter control?

- Clean catch
- Sterile bag applied at perineal level
- Bladder catheter
- Suprapubic puncture

**Result**

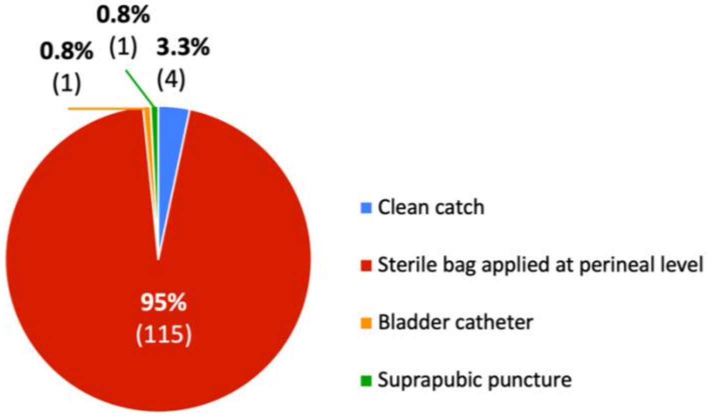


**9)** Which of the following features are considered for the diagnosis of an upper urinary tract infection in a febrile child?

- Leukocytes and nitrites only
- The presence, in the absence of leukocytes or nitrites, of other abnormalities (e.g. proteins or erythrocytes)
- Leukocytes only
- Nitrites only

**Result**

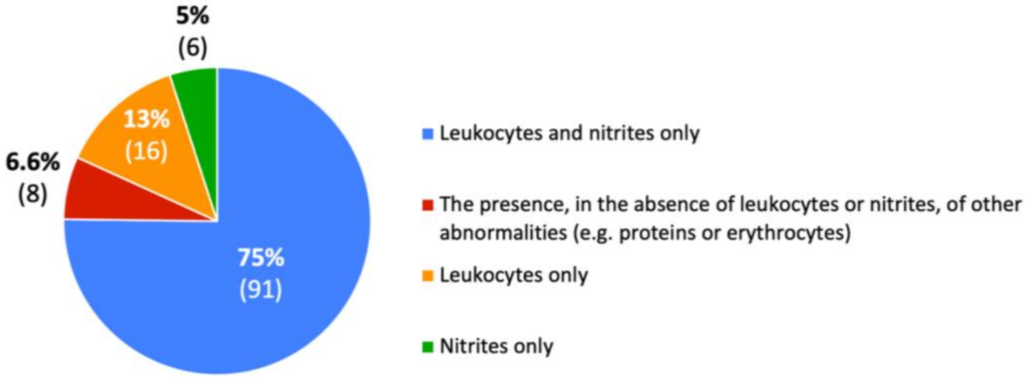


**10)** Is a microscopic urinalysis performed in the workout of a suspected urinary tract infection?

- Never
- <50%
- ≥ 50%
- Always

**Result**

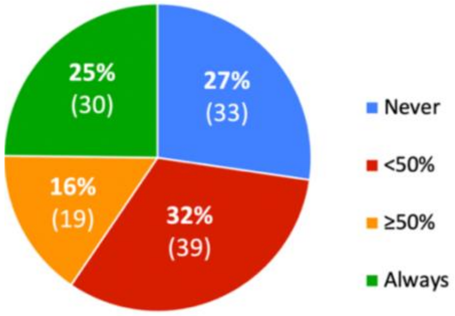


11) Which of these tools is most often used to collect urine to perform a urine culture in a child in a good general condition with a suspected clinical picture of urinary tract infection and without sphincter control?

- Clean catch
- Sterile bag applied at perineal level
- Bladder catheter
- Suprapubic aspiration

**Result**

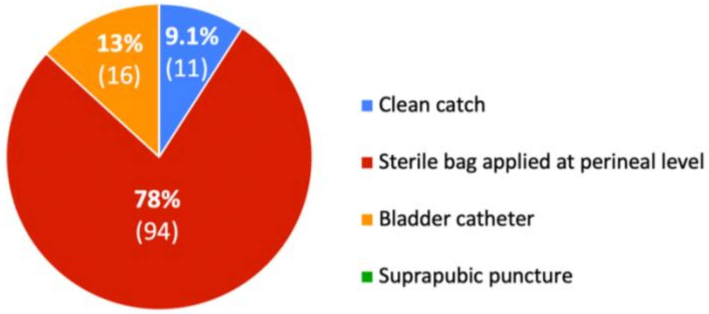


**12a)** If a urine culture from perineal bag is requested and this is positive for a bacterium, what extent of the bacterial load (colony-forming units / CFU) is considered diagnostic for a urinary tract infection?

- 1,000 CFU / mL
- 10,000 CFU / mL
- 50,000 CFU / mL
- 100,000 CFU / mL
- The number of UFCs is not considered relevant

**Result**
2 (1.7%) 1,000 CFU / mL; 2 (1.7%) 10,000 CFU / mL; 2 (1.7%) 50,000 CFU / mL: 1 (0.8%) 50,000 CFU / mL; 114 (94%) 100,000 CFU / mL; 0 (0.0%) The number of UFCs is not considered relevant.

**12b)** If a urine culture from a bladder catheter is requested and this is positive for a bacterium, which entity of the bacterial load (colony-forming units / CFU) is considered diagnostic for a urinary tract infection?

- 1,000 CFU / mL
- 10,000 CFU / mL
- 50,000 CFU / mL
- 100,000 CFU / mL
- The number of UFCs is not considered relevant

**Result**
13 (11%) 1,000 CFU / mL; 58 (48%) 10,000 CFU / mL; 21 (17%) 50,000 CFU / mL: 1 (0.8%) 50,000 CFU / mL; 16 (13%) 100,000 CFU / mL; 13 (11%) The number of UFCs is not considered relevant.

**13)** Are blood tests (e.g. blood cell count, inflammatory markers, electrolytes and renal function) required in a febrile child in a good general condition with a clinical picture and a chemical-physical examination of the urine (urinary stick) consistent with a urinary tract infection?

- Never
- <50%
- ≥ 50%
- Always

**Result**

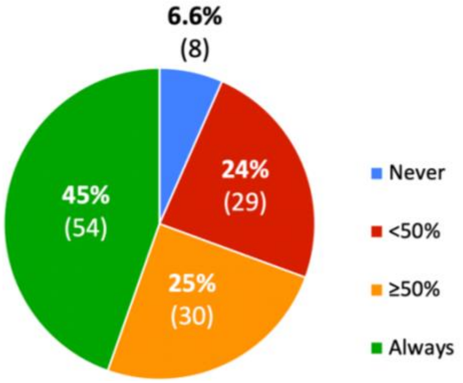


**14)** When is an antimicrobial treatment prescribed in a febrile child in a good general condition with a clinical picture and a urinary dipstick consistent with a urinary tract infection?

- Immediately and without further investigation
- Immediately after collecting the urine for urine culture
- Only if the urine culture is positive
- At least 48 hours after the onset of fever

**Result**

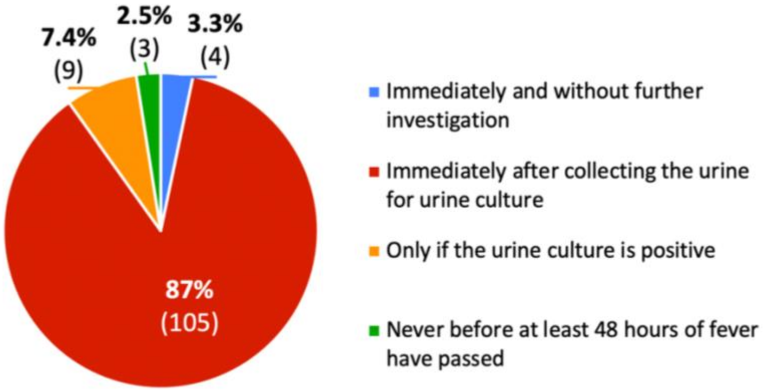


**15)** What empirical oral therapy is prescribed in a febrile child in a good general condition with a urinary tract infection, without known malformations or risk factors for bacterial resistance?

- Amoxicillin
- Amoxicillin + Clavulanic Acid
- Cefuroxime
- Cefixime
- Other: …………………………

**Result**

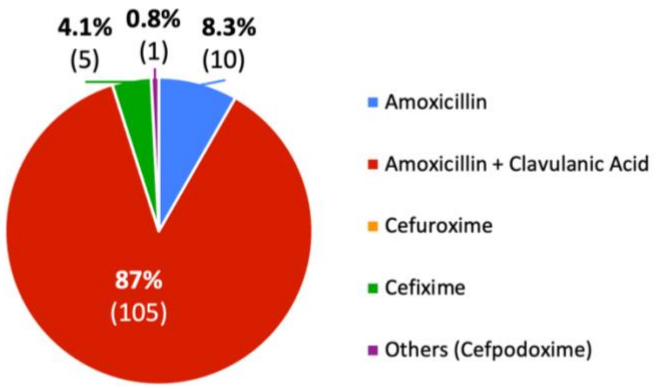


**16)** At what dosage? Specify dosage in mg / kg body weight per day

**Result**
Amoxicillin:
- 9 (7.7%) 80-100 mg/kg body weight
- 1 (0.8%) 80-100 mg/kg body weight
- 1 (0.8%) 100 mg/kg body weight

Amoxicillin + Clavulanic Acid:
- 1 (0.8%) 150 mg/kg body weight
- 2 (1.7%) 100 mg/kg body weight
- 100 (83%) 50-90 mg/kg body weight
- 1 (0.8%) 40 mg/kg body weight

Cefixime:
- 5 (4.1%) 8 mg/kg body weight

Cefpodoxime:
- 1 (0.8%) 10 mg/kg body weight

**17)** Is a kidney and urinary tract ultrasound performed in a febrile child in a good condition with a first episode of urinary tract infection?

- Never
- Only if the child has not undergone prenatal ultrasounds
- Only if prenatal ultrasound showed urinary tract abnormalities
- Only if the urinary tract infection is caused by an atypical germ that is different from *E. coli*
- Always

**Result**
3 (2.5%) Never; 7 (58%) Only if the child has not undergone prenatal ultrasounds;22 (18) Only if prenatal ultrasound showed urinary tract abnormalities; 17 (14%) Only if the urinary tract infection is caused by an atypical germ that is different from *E. coli;* 72 (60%) Always

**18)** When is a urinary tract ultrasound usually requested?

- Immediately or in any case as soon as possible
- Within 48-72 hours
- In a week
- Before a month
- After a month

**Result**
25 (21%) Immediately or in any case as soon as possible; 22 (19%) Within 48-72 hours; 13 (11%) In a week
33 (39%) Before a month; 19 (16%) After a month

**19)** Which of these criteria are used to hospitalize a child with urinary tract infection in a good general condition? (It is possible to indicate more than one answer)

- Expected poor therapeutic adherence
- Age <12 months
- Age <6 months
- Fever with chills
- Child on antibiotic prophylaxis for previous urinary tract infections
- Persistence of fever despite 3-day course of an appropriate antibiotic treatment

**Result**
94 (78%) Expected poor therapeutic adherence; 22 (18) Age <12 months; 72 (59%) Age <6 months: 59 (48%) fever with chills; 54 (45%) Child on antibiotic prophylaxis for previous urinary tract infections; 101 (84%) Persistence of fever despite 3-day course of an appropriate antibiotic treatment
